# Polygenic risk and pleiotropy in neurodegenerative diseases

**DOI:** 10.1016/j.nbd.2020.104953

**Published:** 2020-08

**Authors:** Eftychia Bellou, Joshua Stevenson-Hoare, Valentina Escott-Price

**Affiliations:** Dementia Research Institute, Cardiff University, UK

**Keywords:** Polygenic risk score, Pleiotropy, Heritability, Neurodegenerative disease

## Abstract

In this paper we explore the phenomenon of pleiotropy in neurodegenerative diseases, focusing on Alzheimer's disease (AD). We summarize the various techniques developed to investigate pleiotropy among traits, elaborating in the polygenic risk scores (PRS) analysis. PRS was designed to assess a cumulative effect of a large number of SNPs for association with a disease and, later for disease risk prediction. Since genetic predictions rely on heritability, we discuss SNP-based heritability from genome-wide association studies and its contribution to the prediction accuracy of PRS. We review work examining pleiotropy in neurodegenerative diseases and related phenotypes and biomarkers. We conclude that the exploitation of pleiotropy may aid in the identification of novel genes and provide further insights in the disease mechanisms, and along with PRS analysis, may be advantageous for precision medicine.

## 1 Introduction

Neurodegenerative diseases are a group of disorders that are characterised by the progressive loss of the structure and function of the central nervous system. Examples of neurodegenerative disorders include Alzheimer's disease (AD), Dementia with Lewy bodies (DLB), Frontotemporal lobar degeneration (FTLD) with frontotemporal dementia (FTD) being one of its subgroups, Parkinson's disease (PD), and Amyotrophic lateral sclerosis (ALS). These diseases are heterogeneous in their pathophysiology; although they often present overlapping phenotypes (Gitler et al., 2017).

Fifty million people have dementia world-wide, with 10 million new diagnoses each year (Collaborators, 2019). AD is the most common form (60–70%) of dementia. The fully penetrant mutations in *APP*, *PSEN1* and *PSEN2* genes explain only 1% of AD (Hardy and Selkoe, 2002), whereas common forms of AD have heritability estimates of 0.58–0.79 (Gatz et al., 2006). The field of AD genetic research has now produced extensive evidence that other genes may contribute to disease development in AD. The *APOE* gene on chromosome 19 remains the strongest genetic risk factor associated with the common late-onset form of AD (Kunkle et al., 2019; Saunders et al., 1993). Genome-wide association studies (GWAS) have shown to be the most successful approach in identifying the genetic underpinnings of common forms of AD. Since 2009, nearly 40 risk loci have been identified which have been found to associate with AD at the genome-wide level of significance (Jansen et al., 2019; Kunkle et al., 2019; Lambert et al., 2013; Marioni et al., 2018). Although *APOE* is the strongest predictor of late onset AD, the genetic SNP-based heritability explained by this locus is not high (0.05) (Escott-Price et al., 2017b) compared to genome-wide estimates (0.24–0.53) (Lee et al., 2013a; Ridge et al., 2016; Ridge et al., 2013). DLB is the second most common form of dementia, accounting for 1 in 7 post-mortem diagnoses (Vann Jones and O'Brien, 2014). DLB shows strong evidence that genes play a significant role in disease development (SNP-based heritability estimates: 0.31–0.6 (Guerreiro et al., 2016; Guerreiro et al., 2019). FTD is the second most common form of young-onset dementia after Alzheimer's Disease (AD) (Ratnavalli et al., 2002). FTLD can also co-occur with motor neuron disease (FTD-MND, or FTD-ALS) in a continuous spectrum of phenotypes (Strong et al., 2017). In a study by Rohrer et al., 10% of patients with FTLD had an autosomal dominant history and heritability varied between the different clinical syndromes with behavioural variant frontotemporal dementia being the most heritable (Rohrer et al., 2009). PD is a common neurodegenerative movement disorder, affecting 1–2% of the population over the age of 60. PD patients are affected by different combinations of motor and non-motor symptoms, e.g. tremor, rigidity, fatigue, loss of smell. In the most recent GWAS, it was shown that genetic factors that are common in the population make a substantial contribution to PD, with heritability estimates of 0.16–0.36 explained by common variants (Goldman et al., 2019; Nalls et al., 2019). Finally, ALS is a progressive neurodegenerative disorder of motor neurons caused by the interplay of environmental and genetic factors acted on by time (Al-Chalabi and Hardiman, 2013) with total heritability estimates 0.76 from twin studies (Al-Chalabi et al., 2010), and 0.40–0.60 in family studies (Wingo et al., 2011), whereas the SNP-based heritability is estimated 0.21 (Keller et al., 2014).

Recent studies have shown that different complex human traits are genetically correlated, including disorders of the brain (Anttila et al., 2018). This correlation may in part be explained by a phenomenon, formally known as “pleiotropy” (Sivakumaran et al., 2011). Pleiotropy occurs when a genetic locus affects more than one trait and it is one possible underlying cause for an observed cross-phenotype association (Solovieff et al., 2013). Since neurodegenerative conditions are highly comorbid, with certain pairs of neurodegenerative diseases being genetically more similar than others, the joint analysis of related phenotypes has the potential to uncover additional associations. The pleotropic loci may also point to shared biological mechanisms, which may help to filter out spurious associations by reducing the noise and thus improving prediction accuracy. Importantly, highly pleiotropic SNPs are more likely to be genic (exonic or intronic), and less likely to be tissue specific (Watanabe et al., 2019). Moreover, pleiotropic loci have the potential to serve as targets for interventions that simultaneously prevent or treat multiple diseases. Various methods have been developed that exploit pleiotropic effects and several studies have been conducted in an effort to identify novel genetic associations in neurological disorders.

Despite these GWASs being conducted and hundreds of genomic regions being implicated in various neurodegeneration related traits, these findings have not been translated into clinically useful risk prediction models. For precision disease prevention and treatment, GWAS associated Single Nucleotide Polymorphisms (SNPs) typically account for only a small fraction of the total heritability and thus, cannot provide satisfactory prediction accuracy (Manolio et al., 2009). It is now established that the genetic architecture of most neurodegenerative disorders is highly polygenic (Escott-Price et al., 2015b; Ibanez et al., 2019; Nalls et al., 2019)

The polygenic risk score (PRS) offers a calculation of genetic risk for a disease or a trait based not solely on genome-wide significant SNPs, but on all nominally associated variants (typically thousands of variants). A PRS for a trait is defined as the weighted sum of risk variants, where the weights associated with them are usually taken from a powerful GWAS for that trait/disease. Therefore, the PRS accounts for the small effects of a large number of SNPs which still contribute to disease risk, successfully capturing the polygenicity of a disease. PRS has been widely used for identifying and/or predicting an individual's disease risk (Escott-Price et al., 2015a; Escott-Price et al., 2015b; Ibanez et al., 2017) and for studying genetic overlap between disorders traits (Chaudhury et al., 2019; Creese et al., 2019; McLaughlin et al., 2017).

In this review we sought to 1) to summarize the statistical techniques that can be used to identify pleotropic genes and regions, 2) to discuss the biological mechanisms that are common between neurodegenerative disorders, 3) to explain how heritability estimates are related to the prediction accuracy by the PRS and 4) to explore how PRS analysis can be utilised to model the genetic risk of pleiotropic regions for prediction of shared sub-phenotypes in neurodegeneration.

## 2 Approaches to investigate pleiotropy

Various methods have been developed to assess the common underpinnings among diseases in both genome-wide and regional level, as well as in single-variant level. These multi-trait approaches vary considerably in the statistical techniques they employ and the study design considerations they are based on, including the type and number of traits and the type of data needed (individual-level genotype or summary statistics data).

### 2.1 Genome-wide and regional approaches

Genetic correlation between traits on the genome-wide scale can be used as an initial assessment of the global genetic overlap between two traits. The latter can be inferred by applying the PRS method to two trait GWAS datasets (Purcell et al., 2009). This method uses markers selected from the summary GWAS data in one sample to construct a weighted genetic risk score for each individual in an independent sample. An association between this composite score and the trait of interest in the second sample is evidence of an overlap between the polygenic architecture of each trait.

In like manner, there are a number of multivariate methods for genetic correlation analysis GCTA (Lee et al., 2013b), BOLT-REML (Loh et al., 2015) and mvLMM (Furlotte and Eskin, 2015)) which use individual-level genotype data, limiting their usage due to restrictions around data sharing.

More recently, the LD score regression approach (LDSC) (Bulik-Sullivan et al., 2015) has gained popularity for the investigation of genetic correlation between traits, as only summary statistics data are required for this analysis. This approach is also easy to use and very indicative of potential pleiotropy between traits, but it does not provide information at an individual SNP level. Finally, a recently published method, GNOVA (Lu et al., 2017), was developed to allow the stratification of genetic covariance by functional genome annotation, enabling the investigation of the shared genetic basic between complex phenotypes. It should be noted that genetic correlation is not the same as pleiotropy; zero correlation does not indicate the absence of common risk loci between two traits as there could be lack of directionality to the genetic relationship (i.e. at some shared loci the risk allele is the same for both traits, whereas at others the same allele can increase the risk for one trait but be protective for the other). A technique addressing the latter has been proposed named ρ-HESS (Shi et al., 2017), that finds specific regions with strong correlation that could serve as putative causal models between traits.

### 2.2 Single-variant approaches

In the signal-variant level, the simplest approaches which are commonly used to explore association of genetic variants with multiple correlated phenotypes are 1) examination of single-trait summary statistics and reporting cross-phenotype associations for loci reaching the significance threshold for each trait, and 2) testing loci known to be associated with a given phenotype for association with another phenotype i.e. reporting loci reaching the significance threshold for the second phenotype (Desikan et al., 2015). These approaches can be underpowered, thus various methods that statistically combine summary data for multiple traits have been developed.

Meta-analysis methods combining GWAS summary statistics via conventional inverse-variance meta-analysis increase power but also pose methodological challenges when different studies are capturing heterogeneous and/or pleiotropic phenotypes. In the case of pleiotropy, individual variants are likely to be associated with only a subset of the traits analysed, or they might even demonstrate effects in different directions for the different phenotypes under analysis. Generalisations and modifications of the meta-analyses for the discovery of pleotropic single-variants include the Cross Phenotype Meta-Analysis (CPMA) (Cotsapas et al., 2011), the Association analysis based on SubSETs (ASSET) (Bhattacharjee et al., 2012), the Cross Phenotype ASSOCiation (CPASSOC) (Zhu et al., 2015), the R package MultiMeta (Vuckovic et al., 2015) and the Multi-Trait analysis of GWAS (MTAG) (Turley et al., 2018). CPMA tests whether a SNP has multiple phenotypic associations across different traits that might have a common genetic background, such as autoimmune diseases. It can detect variants that are associated to at least a subset of, though not necessarily all, diseases by examining the deviation in the distribution of associations; however, this method cannot be applied to studies with sample overlap (Cotsapas et al., 2011). On the contrary, ASSET, CPASSOC and MTAG are robust to sharing the same controls, which is essential when summary statistics data come from large consortia. The ASSET technique explores all possible subsets of traits with non-null associations to identify the one with the maximum *Z*-statistic and evaluate its significance while accounting for multiple testing. This method allows a subset of traits to have no effect, or for the effect of the susceptibility loci to manifest in different directions (Bhattacharjee et al., 2012). The MTAG approach is based on the key assumption that all variants have the same effect sizes across the traits and produces trait-specific association statistics. It can be specifically useful for a trait that is underpowered but shows strong genetic correlation with other traits. However, the application of MTAG to a large number of low-powered studies could cause large inflations to the False Discovery Rate (FDR) (Turley et al., 2018). CPASSOC was developed to work for both univariate and multivariate summary statistics data and it allows for heterogeneity of effects for the same or different phenotypes across studies (Zhu et al., 2015). Finally, the R package MultiMeta is applied to a multivariate setting by allowing different effect estimates (weights) for each marker (Vuckovic et al., 2015).

Extending the empirical Bayesian false discovery rate (FDR) (Efron and Tibshirani, 2002), the Bayesian conditional FDR (cFDR) approach is based on the notion that if two diseases share a common genetic background, a degree of association with one trait may increase the likelihood of detecting an association with the second trait (Andreassen et al., 2013). The Mendelian Randomization approach (Smith and Ebrahim, 2004) uses information on the association of one or several SNPs with each trait to infer whether or not trait A causally influences trait B (known as “mediated pleiotropy”). It can be used to detect mediated pleiotropy compared to the above-mentioned methods that aim to detect biological pleiotropy (Solovieff et al., 2013). Finally, BUHMBOX (Han et al., 2016) is aimed at detecting spurious pleiotropy by examining whether alleles with shared risk are observed due to sharing among all individuals or a subset of individuals, in a genetically heterogeneous cohort.

## 3 Risk prediction in neurodegeneration with polygenic risk scores

The genetic architecture of the majority of neurodegenerative diseases includes many common variants of small effect that are likely to reflect a large number of susceptibility genes and a complex set of biological pathways related to disease. While the polygenic method introduces noise by including some variants that are not involved in disease susceptibility (i.e. false positives), this has been shown to be offset by the increased power to identify those at highest/lowest risk of disease (Consortium, 2014; Escott-Price et al., 2015b).

PRS analysis has recently shown that there is a significant polygenic contribution to neurodegenerative diseases. A large polygenic contribution to the overall heritable risk of AD has been reported by (Escott-Price et al., 2015b). Since the whole genome captures a much higher proportion of genetic variability (0.24–0.53) than *APOE* alone (0.05–0.13) (Escott-Price et al., 2017b; Lee et al., 2013a; Ridge et al., 2016), the prediction accuracy using PRS is higher, with an of AUC = 75%–84% in clinical and pathology confirmed samples respectively (Escott-Price et al., 2017a; Escott-Price et al., 2015b). When *APOE* is included in the PRS, the majority of the people at the high extreme of the PRS distribution possess ε4 allele(s), however the predictive accuracy of PRS in pathologically confirmed ε3 homozygotes is also high and is equivalent to the predictive accuracy of the whole dataset (Escott-Price et al., 2019).

In PD, a polygenic basis has been confirmed and shown to correlate with age at disease onset (Escott-Price et al., 2015a). However, the PRS analysis requires large discovery sample sizes to estimate the effect sizes of risk alleles as accurately as possible. The GWAS sample sizes for FTD, DLB, ALS are not as large as AD or PD, and therefore no PRS has been attempted for these diseases (Ibanez et al., 2019). In addition, diseases like FTD and ALS are very heterogeneous and can be stratified into different subtypes, reducing the sample size for each group and thus the power of the GWAS and the PRS analyses.

A common pitfall is to directly compare prediction accuracy by PRS and known strongly associated regions/genes (e.g. *APOE*) with the heritability estimates. Two main measures (*R*^2^ - the proportion of variance explained and AUC - Area Under the receiver operator Curve) are routinely used to report the quality of prediction by the PRS. *R*^2^ is directly linked to the heritability captured by SNPs and is usually quite small.

To illustrate this, we use AD as an example. For AD the *R*^2^ is 0.16 for *APOE* alone and 0.1 for the PRS excluding APOE locus (in house analysis in GERAD data (Baker et al., 2019) using summary statistics from (Kunkle et al., 2019)). AUC is the accuracy of the prediction, comparing the observed case/control status and the predicted classification estimated by a logistic regression model. It provides an aggregate measure of performance across all possible classification thresholds. An AUC of 50% indicates that the model cannot discriminate between cases and controls. For AD, the AUC is 67% for *APOE* alone and 63% for PRS without *APOE* (Escott-Price et al., 2015b). When the two variables (*APOE* and PRS) are combined, the AUC for the joint prediction is 75%.

An explanation for this can be found in (Wray et al., 2019). The expected value of *R*^2^ (*E*(*R*^2^)) is the SNP-based heritability (*h*^2^) divided by the sum of one plus the ratio of the number of SNPs (*M*) by the sample size (*N*) times *h*^2^)

$$E\left(R^{2}\right)=\frac{h^{2}}{1+M/\left(Nh^{2}\right)}.$$

The SNP-based heritability is a value between 0 and 1. If *M* is small and *N* is large, then the ratio *M*/(*Nh*^2^) tends to zero, and *E(R*^2^) approximately equals the heritability (*h*^2^). However, when the number of SNPs is large and comparable with the sample size (e.g. PRS including ~87 K SNPs (Escott-Price et al., 2015b), *M* ~ 74 K in the International Genomics of Alzheimer's Project (IGAP) (Lambert et al., 2013)), the ratio *M*/(*Nh*^2^) is not small. Therefore, the *R*^2^ value is (much) smaller than the SNP-based heritability. For example, the expected *E*(*R*^2^) ~ 0.04 for AD (using *M* and *N* from the example above and *h*^2^ = 0.24), which is similar to the results reported in (Escott-Price et al., 2015b). Thus, the *APOE* locus alone has both a higher *R*^2^ and AUC than a PRS without *APOE*, whereas the SNP-based heritability explained by the *APOE* region is much lower (0.05–0.13) than that explained by the whole genome (0.24–0.53) (Escott-Price et al., 2017b; Lee et al., 2013a; Ridge et al., 2016).

In addition, there are two types of genetic heritability: *broad* sense and *narrow* sense (the latter is also known as a SNP-based heritability). Narrow sense heritability is the proportion of variation in the trait that can be explained by only *additive* effects of *common* SNPs, so it is almost always less than the total heritability that could be explained by all genetic factors (*broad* sense) (Wray and Visscher, 2008). The *narrow* sense heritability does not account for rare variants, Copy Number Variations (CNVs), SNPxSNP interactions, dominance, etc.

To date, the broad sense heritability was reported for AD (0.58–0.79 (Gatz et al., 2006)), PD (0.27 (Goldman et al., 2019)) and ALS (Al-Chalabi et al., 2010; Wingo et al., 2011), whereas the SNP-based heritability was estimated as 0.24–0.53 (Lee et al., 2013a; Ridge et al., 2016), 0.16–0.26 (Nalls et al., 2019) and 0.21 (Keller et al., 2014), respectively. We emphasise that these are different types of heritability estimates, and there is no inherent contradiction between them. Indeed, taking AD again as an example, Gatz et al. (2006) explore the co-occurrence of clinically diagnosed AD in families with monozygotic and dizygotic twins, and thus estimated the *broad* sense heritability. In their study, the 0.58 heritability estimate has a very broad 95% confident interval (CI) [0.19–0.87], and the 0.79 [95% CI: 0.67–0.88] heritability estimate is obtained using a model where the “shared environmental influences” parameter is dropped (see Table 2 in (Gatz et al., 2006)). The shared component in neurodegenerative disorders may not be as essential to account for as for neurodevelopmental disorders, however in light of known association of e.g. AD PRS with educational attainment (EA) (Hagenaars et al., 2016), it may still be relevant.

SNPs, and in turn PRS, estimate/capture the *narrow* sense heritability. PRS picks up the *narrow* sense heritability in AD quite well (Escott-Price et al., 2017b). To compare (Gatz et al., 2006) and (Lee et al., 2013a) estimates, the difference between “heritability on observed scale” versus “heritability on liability scale” needs to be accounted for. A binary trait (case/control) has to be treated as if it has an underlying continuous liability. PRS has a continuous normal distribution in a population, and only individuals at the right tail of the distribution are likely to develop AD. In case/control studies we estimate “heritability on observed scale”. To project it to the whole population, the “heritability on observed scale” must be converted to the “heritability on liability scale”, accounting for the disease prevalence as follows $h_{\mathrm{Liab}}^{2}=h_{\mathrm{obs}}^{2}\frac{{\left[K\left(1-K\right)\right]}^{2}}{Z^{2}\;P\left(1-P\right)}$, where *h*_*Liab*_^2^ is the heritability estimate on liability scale, *K* is the prevalence of the disease, *P* is the proportion of cases in the study and *h*_*obs*_^2^ is the heritability estimate on observed scale. *Z* is the liability threshold defined by the standard normal distribution depending on the prevalence *K* (see eq. (23) from (Lee et al., 2011)). For AD in case/control data, the (Lee et al., 2013a) approach estimates the *narrow* sense heritability on *liability* scale as 0.24 (assuming a disease prevalence (lifetime risk) of 2% for AD (Brookmeyer et al., 1998). The Gatz et al. approach estimates the *broad* sense heritability on *liability* scale (Gatz et al., 2006) and their sample was 65+ years. The AD prevalence in 65+ age group is 10% (Thies and Bleiler, 2012), so the *h*^2^ on the *liability* scale assuming a disease prevalence (65+) of 10%, is ~0.39. This *narrow* sense heritability on liability scale is, as expected, lower than Gatz et al.'s *broad* sense heritability estimate and falls within its confidence intervals. The same applies to heritability estimates for other reported neurological disorders. The broad sense heritability of PD (0.27 (Goldman et al., 2019)) is within the interval of the reported narrow sense heritability (0.16–0.36 (Nalls et al., 2019)), and for the ALS the estimates are not so close (0.4–0.76 (Al-Chalabi et al., 2010; Wingo et al., 2011) and 0.21 (Keller et al., 2014) for the broad and narrow sense heritability, respectively).

## 4 Pleiotropy in neurodegeneration

Several studies have been conducted in an effort to investigate mostly the genetic overlap between traits in neurological disorders, and consequently identify novel genetic associations.

*Genetic overlap and pleiotropy between neurodegenerative diseases.*

The Brainstorm Consortium, a collaboration among GWAS meta-analysis consortia for 25 disorders, performed a comprehensive heritability and correlation analysis of brain disorders (Anttila et al., 2018). Neurological disorders showed a limited extent of genetic correlation compared to psychiatric disorders, suggesting greater diagnostic specificity and/or more distinct aetiologies. PD, AD, generalised epilepsy, and multiple sclerosis showed little to no correlation with other brain disorders. Interestingly, despite AD and PD being clinically distinct entities, there is pathological evidence of Lewy body deposition (which is central to DLB) in AD that has been reported to be more extensive in familial AD cases and in AD cases with a variant pathology (Lippa et al., 1997). Similarly, an AD-like pathology has been reported in some PD cases, with a correlation being found between cortical amyloid pathology, neurofibrillary tangle pathology, and dementia in PD (Compta et al., 2011). Guerreiro et al. (2016) compared AD and PD summary GWAS statistics using restricted maximum likelihood and obtained a statistically significant but relatively low genetic correlation r_g_ = 0.08 (Guerreiro et al., 2016). This study however showed a significant genetic correlation between AD and DLB (r_g_ = 0.578) and between PD and DLB (r_g_ = 0.362). The former correlation remained substantial (r_g_ = 0.332) even when the *APOE* locus was excluded. The PD-DLB-AD spectrum may share some underlying mechanisms; however, the absence of genetic overlap between AD and PD reported by Guerreiro et al. (2016) might be indicative of different biological pathways underlying the association between DLB and AD and PD. Finally, the authors presenting the GNOVA technique identified a significant correlation between AD and ALS using both their method and LDSC (r_g_ = 0.175 and r_g_ = 0.12, respectively) (Lu et al., 2017). A potential common neuroinflammation pathway, underlying both diseases, was suggested after tissue-stratified analysis, with covariance showing significance in the immune annotation track (*p*-value = .014). The results above show weak to moderate evidence for genetic correlations between neurodegenerative disorders, but do not explicitly indicate the molecular mechanisms of the disease development.

Further attempts were made to identify specific loci and genes indicating pleotropic effects, as the absence of genetic overlap between traits at the genome-wide level does not implicate the absence of pleiotropic genes/SNPs. Although advancing age is a common risk factor for both AD and PD, studies that have investigated the extent to which these two diseases co-occur in families have produced varying results. A study by Moskvina et al. (Moskvina et al., 2013) revealed no significant evidence for the presence of alleles that increase the risk of both diseases. The authors of another study (Desikan et al., 2015) reported genetic overlap between AD and PD at the tau-associate *MAPT* locus (variant rs393152) and conducted an enrichment analysis in AD as a function of significance in PD with cFDR with and without *MAPT* locus. Removing *MAPT,* there was a considerable attenuation of enrichment, so the observed pleiotropy was non-polygenic and confined to the *MAPT* region. The reported variant, that tags the H1 haplotype at *MAPT* locus which has been associated with various tauopathies (Pittman et al., 2006), was also significantly associated with longitudinal medial temporal lobe atrophy. Finally, a study assessed the genetic overlap between frontotemporal dementia (FTD), AD and PD using conjunction cFDR and identified novel FTD risk associated markers using cFDR (Ferrari et al., 2017). Polygenic enrichment was observed for FTD SNPs conditional on AD and PD across different significance levels providing evidence of pleiotropy. A similar pattern was observed for AD SNPs and PD SNPs conditional on FTD. Moreover, eight loci were found to be FTD-PD specific, one to be FTD-AD specific and 13 novel FTD associations were identified within the *HLA*, *MAPT* and *APOE* regions. The findings support the role of immune response, lysosomal processes, intracellular vesicular trafficking and chromatin metabolism in neurodegenerative diseases (Ferrari et al., 2017).

### 4.1 Genetic overlap and pleiotropy between neurodegenerative and neurodegenerative-associated traits

A number of studies have investigated phenotypic links between AD and known associated phenotypes including EA, cognitive impairment and neuropathological traits. Using 112,151 participants of the UK Biobank, the pleiotropic effects between cognitive functioning and AD were quantified using both LDSC and PRS analysis (Hagenaars et al., 2016). Significant negative correlations were found with verbal-numerical reasoning (r_g_ = −0.39) and with EA (r_g_ = −0.27), respectively. The PRS analysis replicated the latter results; higher polygenic risk for AD was associated with lower score on verbal-numerical reasoning (size effect β = −0.023, *p* = 1.27 × 10^−5^), more errors on a short-term recall task (β = −0.011, *p* = 1.22 × 10^−4^), and lower EA (β = −0.046, *p* = 2.33 × 10^−12^). Similar results were reported in a similar study using LDSC using data from the CHARGE consortium (Hill et al., 2016). AD was negatively correlated with both childhood and old age general cognitive function (r_g_ = −0.341, *p* = .001 and r_g_ = −0.324, *p* = 1.78 × 10^−5^, respectively), and with EA (r_g_ = −0.324, *p* = 1.15 × 10^−5^). Lu et al. (2017) jointly analysed AD and other 49 traits and reported significant negative correlations between cognition and EA. No significant correlations were found between AD and trait neuroticism or diseases like depression and schizophrenia (Lu et al., 2017). In 2018, Hagenaars et al. repeated their analyses using the UK Biobank, focusing on the overlap of AD, ALS and FTD with cognitive ability and physical function (Hagenaars et al., 2018). PRS for AD significantly predicted, as before, verbal-numerical reasoning and short-term recall. When the *APOE* region was excluded, the results remained significant. Moreover, higher ALS PRS was significantly associated with answering fewer verbal-reasoning questions correctly (β = −0.019). In a meta-analysis study for general cognition function, SNPs in a number of genes were identified which have also been implicated in Alzheimer's disease, including *SLC39A1* (Olesen et al., 2016), *TTBK1* (Ikezu and Ikezu, 2014), *MAPT*, *WNT3*, *CRHR1*, *KANSL1*, and *NSF* (Jun et al., 2016). Furthermore, using LDSC the estimated genetic correlation between AD and cognition was found to be r_g_ = −0.37 with *p* = 2.78 × 10^−5^ (Davies et al., 2018). The protective relationship between EA and AD was replicated using Mendelian randomization using 1271 SNPs (odds ratio = 0.63, 95% CI: 0.54–0.74; *p* = 4.08 × 10^−8^) (Raghavan et al., 2019), and the regions that replicated the causal relationship were found to contain genes involved in the regulation of the neural development. The common underpinnings of cognition and health measures support the theoretical hypothesis of bodily system integrity (Deary, 2012). The latter is based on the idea that there is a latent trait of well-functioning body with, for example, higher levels of cognitive function being one aspect of that body that can respond well to environmental challenges and be resistant to disease.

Chung et al. (2018) conducted genome-wide pleiotropy analyses using GWAS summary statistics for AD-related neuropathological traits, including neuritic plaque (NP), neurofibrillary tangle (NFT), and cerebral amyloid angiopathy (CAA). Genome-wide significant pleiotropic associations were observed for a single SNP in the joint models of NPs and NFTs; and for NFTs and CAA at SNP and gene-based levels (Chung et al., 2018). Both identified regions, *C20orf40* and *HDAC9*, showed reduced expression in subjects with AD compared to controls in several brain regions. The authors also suggested that *HDAC9* along with *MEF2C*, a well-established AD risk loci (Lambert et al., 2013), might participate in a pathway leading to the formation of neurofibrillary tangles and brain atrophy (Chung et al., 2018). Finally, in a recent study the overlap between brain age gap and brain disorders was assessed (Kaufmann et al., 2019). No genetic correlation estimated with LDSC was significant after applying FDR correction for multiple testing; however, when using cFDR, six significant independent loci were found showing genetic overlap between brain age gaps and AD.

### 4.2 Genetic overlap and pleiotropy between neurodegenerative and non-degenerative diseases

There have also been a number of studies showing genetic overlap between AD and non-neurodegenerative disorders; in particular AD and bipolar disorder (BIP), implicating the *MARK2* and *VAC14* genes using cFDR analysis (Drange et al., 2019), AD and breast and lung cancer using LDSC (r_g_ = 0.18, *p* = .03 and r_g_ = 0.30, *p* = .01, respectively) (Feng et al., 2017), and AD family history and depression, although this showed a non-significant correlation (Gibson et al., 2017). Both *MARK2* and *VAC14*, jointly involved in the genetic aetiology of AD and BIP, have been described to play a role in neuronal migration, tau phosphorylation (Gu et al., 2013; Matenia and Mandelkow, 2009; Reiner et al., 2009) and endosomal homeostasis (Di Paolo and De Camilli, 2006), whereas the regulation of gene expression in relation to enhancer activity might play a crucial role in the shared heritability of AD and cancer (Feng et al., 2017). In a study conducted to explore the share genetics between AD and cardiovascular disease (CAD), and additionally between their shared risk factors and each disease (Karlsson et al., 2017), the authors using polygenic scores found no association between CAD PRS and dementia after controlling for age, sex, education, and diabetes and no common cluster of significant genes for AD and CAD. AD and CAD each had a significant number of genes in common with low density lipoprotein cholesterol and total cholesterol, but not with the same genes, whereas only AD overlapped with high-density lipoprotein cholesterol and triglycerides. Additionally, AD and Body Mass Index (BMI) were found to have a significant number of shared genes, whereas no genetic overlap was found between AD and Type 2 Diabetes (T2D). Similarly, Proitsi et al. found no association between the PRSs based on T2D and the increased risk of AD (Proitsi et al., 2014). It has been suggested that beta amyloid plaques and metabolic changes precede dementia (Jack et al., 2009), thus T2D might be a metabolic consequence of AD.

Broce et al. (2019) studied the shared genetic variance between AD and CVD associated risk factors including BMI, T2D, CAD, waist hip ratio (WHR), lipid levels like high-density lipoprotein (HDL) and low-density lipoprotein (LDL), total cholesterol (TC), and triglycerides (TG) (Broce et al., 2019). Using the cFDR approach, they reported a pleiotropic genetic enrichment of significant SNPs for the plasma lipid levels and 90 SNPs that are jointly associated with increased risk of AD and CVD outcomes. High plasma lipid levels are thought to possibly lead to pathological cholesterol metabolism in the brain (Xue-Shan et al., 2016). Finally, Lin et al. (2019) used AD PRS to examine the polygenic overlap between AD and vascular pathologies including lobar cerebral microbleeds (CMB), white matter lesions (WML), retinal venular diameter, carotid intima-media thickness and coronary artery classification (CAC). Their findings were mostly due to *APOE*, which showed an association with CMB, WML and CAC, and two cognition outcomes (Lin et al., 2019).

Investigating the relationship between PD and autoimmunity using cFDR and conjunction cFDR (Witoelar et al., 2017), 17 novel genetic variants were found to be common with type 1 diabetes, Crohn's disease, ulcerative colitis, rheumatoid arthritis, celiac disease, psoriasis, and multiple sclerosis, suggesting that the immune system affects the PD aetiology. Since then, a positive and significant correlation has been found between PD and PD-inferred gene expression and melanoma (r_g_ = 0.17 and r_g_ = 0.14, respectively) (Dube et al., 2019).

## 5 Discussion

The existence of pleiotropy suggests that common pathological mechanisms may underlie neurodegenerative disorders. While neurodegenerative diseases have distinct pathologies, there are also shared pathological features like protein aggregation in the brain. It has also become apparent that common pathways exist in the pathogenesis of neurodegeneration: aberrant ion channel function, mitochondrial dysfunction, defects in intracellular trafficking and axonal transport, abnormal protein aggregation and clearance (Hashimoto et al., 2018). In addition, the association between inflammation and neurodegenerative diseases has long been observed in AD, ALS and PD (Witoelar et al., 2017). Given the various molecular mechanisms that can drive the association of the shared genetic risk variants, the biological interpretation of pleiotropy is challenging. With the increased availability of GWAS summary statistics over the last decade, the phenomenon of pleiotropy and its exploitation has received increasing attention as pleiotropy may inform reasons for comorbidity between traits, pointing to underlying shared biological pathways, and thus may aid in orienting the causality between traits.

Medical treatments and interventions for neurodegenerative diseases typically use a ‘one-size-fits-all’ approach, in which broad treatment programmes are recommended to all patients. Given the complex and heterogeneous nature of diseases such as AD, there is great potential to improve these treatments though the use of precision medicine. Precision medicine is an approach whereby the individual characteristics of a patient, their specific genotype or phenotype, and progression stage of the disease are taken into account to suggest the most appropriate medical treatment. This approach is widely used, for example, in oncology (Garraway et al., 2013). Although broadly thought of in the public domain as one entity, cancers exhibit a range of aetiologies, progressions, and presentations depending on their type. Thus, different medical treatments are provided dependent on the type and stage of cancer, and the characteristics of the patient being treated. Application of similar methods in Alzheimer's disease would better represent the varied nature of this and similar dementias and neurodegenerative diseases.

At present the largest obstacle for precision medicine approaches in neurodegenerative disorders is the relative infancy of research into the causes of these disorders, in particular at a molecular level. Polygenic risk scores as a tool to model pleiotropic loci for disease and its sub-phenotypes have a great potential to contribute to precision medicine for neurodegenerative disorders. As GWASs examine the entire genome, in combination with pleiotropy they have the ability to highlight previously unknown genes, and to examine their association with a number of phenotypes directly related to the disease and/or phenotypes which are consequences of disease development and progression.

In conclusion, the molecular mechanisms underlying pleiotropy is likely to be diverse and understanding of these mechanisms is vital for understanding pathogenesis of neurodegenerative disorders. Pleiotropy in combination with the risk prediction utility of PRS, this can form the foundation stones upon which precision medicine for neurodegenerative disorders is built. Hence, research utilising these approaches is crucial for initiatives aiming to bring precision medicine to the field of neurodegenerative disease. The associations between pathway specific PRS and phenotypic changes, accounting for insights from pleiotropy, may allow us to define the biology of disease in individuals, heralding precision medicine in neurodegeneration.

## Declaration of Competing Interest

The authors EB, VEP have collaborated with Cytox Ltd. through their InnovateUK grant funding.

## References

[bb0005] Al-Chalabi A., Hardiman O. (2013). The epidemiology of ALS: a conspiracy of genes, environment and time. Nat. Rev. Neurol..

[bb0010] Al-Chalabi A. (2010). An estimate of amyotrophic lateral sclerosis heritability using twin data. J. Neurol. Neurosurg. Psychiatry.

[bb0015] Andreassen O.A. (2013). Improved detection of common variants associated with schizophrenia and bipolar disorder using pleiotropy-informed conditional false discovery rate. PLoS Genet..

[bb0020] Anttila V. (2018). Analysis of shared heritability in common disorders of the brain. Science..

[bb0025] Baker E. (2019). Gene-based analysis in HRC imputed genome wide association data identifies three novel genes for Alzheimer’s disease. PLoS One.

[bb0030] Bhattacharjee S. (2012). A subset-based approach improves power and interpretation for the combined analysis of genetic association studies of heterogeneous traits. Am. J. Hum. Genet..

[bb0035] Broce I.J. (2019). Dissecting the genetic relationship between cardiovascular risk factors and Alzheimer’s disease. Acta Neuropathol..

[bb0040] Brookmeyer R. (1998). Projections of Alzheimer’s disease in the United States and the public health impact of delaying disease onset. Am. J. Public Health.

[bb0045] Bulik-Sullivan B. (2015). An atlas of genetic correlations across human diseases and traits. Nat. Genet..

[bb0050] Chaudhury S. (2019). Alzheimer’s disease polygenic risk score as a predictor of conversion from mild-cognitive impairment. Transl. Psychiatry.

[bb0055] Chung J. (2018). Genome-wide pleiotropy analysis of neuropathological traits related to Alzheimer’s disease. Alzheimers Res. Ther..

[bb0060] Collaborators G.D. (2019). Global, regional, and national burden of Alzheimer’s disease and other dementias, 1990-2016: a systematic analysis for the global burden of disease study 2016. Lancet Neurol..

[bb0065] Compta Y. (2011). Lewy- and Alzheimer-type pathologies in Parkinson's disease dementia: which is more important?. Brain..

[bb0070] Consortium, S. W. G. o. t. P. G (2014). Biological insights from 108 schizophrenia-associated genetic loci. Nature..

[bb0075] Cotsapas C. (2011). Pervasive sharing of genetic effects in autoimmune disease. PLoS Genet..

[bb0080] Creese B. (2019). Examining the association between genetic liability for schizophrenia and psychotic symptoms in Alzheimer’s disease. Transl. Psychiatry.

[bb0085] Davies G. (2018). Study of 300,486 individuals identifies 148 independent genetic loci influencing general cognitive function. Nat. Commun..

[bb0090] Deary I.J. (2012). Looking for ‘system integrity’ in cognitive epidemiology. Gerontology..

[bb0095] Desikan R.S. (2015). Genetic overlap between Alzheimer’s disease and Parkinson’s disease at the MAPT locus. Mol. Psychiatry.

[bb0100] Di Paolo G., De Camilli P. (2006). Phosphoinositides in cell regulation and membrane dynamics. Nature..

[bb0105] Drange O.K. (2019). Genetic overlap between Alzheimer’s disease and bipolar disorder implicates the MARK2 and VAC14 genes. Front. Neurosci..

[bb0110] Dube U. (2019). Overlapping genetic architecture between Parkinson disease and melanoma. Acta Neuropathol..

[bib481] Efron B, Tibshirani R (2002). Empirical Bayes methods and false discovery rates for microarrays.. Genetic Epidemiology.

[bb0115] Escott-Price V. (2015). Polygenic risk of Parkinson disease is correlated with disease age at onset. Ann. Neurol..

[bb0120] Escott-Price V. (2015). Common polygenic variation enhances risk prediction for Alzheimer’s disease. Brain..

[bb0125] Escott-Price V. (2017). Polygenic risk score analysis of pathologically confirmed Alzheimer disease. Ann. Neurol..

[bb0130] Escott-Price V. (2017). Polygenic score prediction captures nearly all common genetic risk for Alzheimer's disease. Neurobiol. Aging.

[bb0135] Escott-Price V. (2019). Polygenic risk score analysis of Alzheimer’s disease in cases without APOE4 or APOE2 alleles. J Prev Alzheimers Dis..

[bb0140] Feng Y.A. (2017). Investigating the genetic relationship between Alzheimer’s disease and cancer using GWAS summary statistics. Hum. Genet..

[bb0145] Ferrari R. (2017). Genetic architecture of sporadic frontotemporal dementia and overlap with Alzheimer’s and Parkinson’s diseases. J. Neurol. Neurosurg. Psychiatry.

[bb0150] Furlotte N.A., Eskin E. (2015). Efficient multiple-trait association and estimation of genetic correlation using the matrix-variate linear mixed model. Genetics..

[bb0155] Garraway L.A. (2013). Precision oncology: an overview. J. Clin. Oncol..

[bb0160] Gatz M. (2006). Role of genes and environments for explaining Alzheimer disease. Arch. Gen. Psychiatry.

[bb0165] Gibson J. (2017). Assessing the presence of shared genetic architecture between Alzheimer’s disease and major depressive disorder using genome-wide association data. Transl. Psychiatry.

[bb0170] Gitler A.D. (2017). Neurodegenerative disease: models, mechanisms, and a new hope. Dis. Model. Mech..

[bb0175] Goldman S.M. (2019). Concordance for Parkinson’s disease in twins: a 20-year update. Ann. Neurol..

[bb0180] Gu G.J. (2013). Elevated MARK2-dependent phosphorylation of tau in Alzheimer’s disease. J. Alzheimers Dis..

[bb0185] Guerreiro, R., et al., 2016. Genome-wide analysis of genetic correlation in dementia with Lewy bodies, Parkinson's and Alzheimer's diseases. Neurobiol. Aging. 38**,** 214.e7-214.e10. 10.1016/j.neurobiolaging.2015.10.028 PMC4759606 26643944

[bb0190] Guerreiro R. (2019). Heritability and genetic variance of dementia with Lewy bodies. Neurobiol. Dis..

[bb0195] Hagenaars S.P. (2016). Shared genetic aetiology between cognitive functions and physical and mental health in UK biobank (N=112 151) and 24 GWAS consortia. Mol. Psychiatry.

[bb0200] Hagenaars S.P. (2018). Genetic risk for neurodegenerative disorders, and its overlap with cognitive ability and physical function. PLoS One.

[bb0205] Han B. (2016). A method to decipher pleiotropy by detecting underlying heterogeneity driven by hidden subgroups applied to autoimmune and neuropsychiatric diseases. Nat. Genet..

[bb0210] Hardy J., Selkoe D.J. (2002). The amyloid hypothesis of Alzheimer’s disease: progress and problems on the road to therapeutics. Science..

[bb0215] Hashimoto M. (2018). Evolvability and neurodegenerative disease: antagonistic Pleiotropy phenomena derived from amyloid aggregates. J. Park. Dis..

[bb0220] Hill W.D. (2016). Age-dependent Pleiotropy between general cognitive function and major psychiatric disorders. Biol. Psychiatry.

[bb0225] Ibanez L. (2017). Parkinson disease polygenic risk score is associated with Parkinson disease status and age at onset but not with alpha-synuclein cerebrospinal fluid levels. BMC Neurol..

[bb0230] Ibanez L. (2019). Polygenic risk scores in neurodegenerative diseases: a review. Curr. Genetic Med. Rep..

[bb0235] Ikezu S., Ikezu T. (2014). Tau-tubulin kinase. Front. Mol. Neurosci..

[bb0240] Jack C.R. (2009). Serial PIB and MRI in normal, mild cognitive impairment and Alzheimer’s disease: implications for sequence of pathological events in Alzheimer’s disease. Brain..

[bb0245] Jansen I.E. (2019). Genome-wide meta-analysis identifies new loci and functional pathways influencing Alzheimer’s disease risk. Nat. Genet..

[bb0250] Jun G. (2016). A novel Alzheimer disease locus located near the gene encoding tau protein. Mol. Psychiatry.

[bb0255] Karlsson I.K. (2017). Genetic susceptibility to cardiovascular disease and risk of dementia. Transl. Psychiatry.

[bb0260] Kaufmann T. (2019). Common brain disorders are associated with heritable patterns of apparent aging of the brain. Nat. Neurosci..

[bb0265] Keller M.F. (2014). Genome-wide analysis of the heritability of amyotrophic lateral sclerosis. JAMA Neurol..

[bb0270] Kunkle B.W. (2019). Genetic meta-analysis of diagnosed Alzheimer’s disease identifies new risk loci and implicates Aβ, tau, immunity and lipid processing. Nat. Genet..

[bb0275] Lambert J.C. (2013). Meta-analysis of 74,046 individuals identifies 11 new susceptibility loci for Alzheimer’s disease. Nat. Genet..

[bb0280] Lee S.H. (2011). Estimating missing heritability for disease from genome-wide association studies. Am. J. Hum. Genet..

[bb0285] Lee S.H. (2013). Estimation and partitioning of polygenic variation captured by common SNPs for Alzheimer’s disease, multiple sclerosis and endometriosis. Hum. Mol. Genet..

[bb0290] Lee S.H. (2013). Genetic relationship between five psychiatric disorders estimated from genome-wide SNPs. Nat. Genet..

[bb0295] Lin Y.F. (2019). Genetic overlap between vascular pathologies and Alzheimer’s dementia and potential causal mechanisms. Alzheimers Dement..

[bb0300] Lippa C.F. (1997). Alzheimer’s disease, Lewy body disease and aging: a comparative study of the perforant pathway. J. Neurol. Sci..

[bb0305] Loh P.R. (2015). Contrasting genetic architectures of schizophrenia and other complex diseases using fast variance-components analysis. Nat. Genet..

[bb0310] Lu Q. (2017). A powerful approach to estimating annotation-stratified genetic covariance via GWAS summary statistics. Am. J. Hum. Genet..

[bb0320] Manolio T.A. (2009). Finding the missing heritability of complex diseases. Nature..

[bb0325] Marioni R.E. (2018). GWAS on family history of Alzheimer’s disease. Transl. Psychiatry.

[bb0330] Matenia D., Mandelkow E.M. (2009). The tau of MARK: a polarized view of the cytoskeleton. Trends Biochem. Sci..

[bb0335] McLaughlin R.L. (2017). Genetic correlation between amyotrophic lateral sclerosis and schizophrenia. Nat. Commun..

[bb0340] Moskvina V. (2013). Analysis of genome-wide association studies of Alzheimer disease and of Parkinson disease to determine if these 2 diseases share a common genetic risk. JAMA Neurol..

[bb0345] Nalls M.A. (2019). Identification of novel risk loci, causal insights, and heritable risk for Parkinson’s disease: a meta-analysis of genome-wide association studies. Lancet Neurol..

[bb0350] Olesen R.H. (2016). Obesity and age-related alterations in the gene expression of zinc-transporter proteins in the human brain. Transl. Psychiatry.

[bb0355] Pittman A.M. (2006). Untangling the tau gene association with neurodegenerative disorders. Hum. Mol. Genet..

[bb0360] Proitsi P. (2014). Alleles that increase risk for type 2 diabetes mellitus are not associated with increased risk for Alzheimer's disease. Neurobiol. Aging.

[bb0365] Purcell S.M. (2009). Common polygenic variation contributes to risk of schizophrenia and bipolar disorder. Nature..

[bb0370] Raghavan N.S. (2019). Genomic variation in educational attainment modifies Alzheimer disease risk. Neurol Genet..

[bb0375] Ratnavalli E. (2002). The prevalence of frontotemporal dementia. Neurology..

[bb0380] Reiner O. (2009). Neuronal migration and neurodegeneration: 2 sides of the same coin. Cereb. Cortex.

[bb0385] Ridge P.G. (2013). Alzheimer’s disease: analyzing the missing heritability. PLoS One.

[bb0390] Ridge P.G. (2016). Assessment of the genetic variance of late-onset Alzheimer’s disease. Neurobiol. Aging.

[bb0395] Rohrer J.D. (2009). The heritability and genetics of frontotemporal lobar degeneration. Neurology..

[bb0400] Saunders A.M. (1993). Association of apolipoprotein E allele epsilon 4 with late-onset familial and sporadic Alzheimer’s disease. Neurology..

[bb0405] Shi H. (2017). Local genetic correlation gives insights into the shared genetic architecture of complex traits. Am. J. Hum. Genet..

[bb0410] Sivakumaran S. (2011). Abundant pleiotropy in human complex diseases and traits. Am. J. Hum. Genet..

[bb0415] Smith G.D., Ebrahim S. (2004). Mendelian randomization: prospects, potentials, and limitations. Int. J. Epidemiol..

[bb0420] Solovieff N. (2013). Pleiotropy in complex traits: challenges and strategies. Nat. Rev. Genet..

[bb0425] Strong M.J. (2017). Amyotrophic lateral sclerosis - frontotemporal spectrum disorder (ALS-FTSD): revised diagnostic criteria. Amyotroph Lateral Scler Frontotemporal Degener..

[bb0430] Thies W., Bleiler L. (2012). 2012 Alzheimer’s disease facts and figures Alzheimer’s Association. Alzheimers Dement..

[bb0435] Turley P. (2018). Multi-trait analysis of genome-wide association summary statistics using MTAG. Nat. Genet..

[bb0440] Vann Jones S.A., O’Brien J.T. (2014). The prevalence and incidence of dementia with Lewy bodies: a systematic review of population and clinical studies. Psychol. Med..

[bb0445] Vuckovic D. (2015). MultiMeta: an R package for meta-analyzing multi-phenotype genome-wide association studies. Bioinformatics..

[bb0450] Watanabe K. (2019). Genetic mapping of cell type specificity for complex traits. Nat. Commun..

[bb0455] Wingo T.S. (2011). The heritability of amyotrophic lateral sclerosis in a clinically ascertained United States research registry. PLoS One.

[bb0460] Witoelar A. (2017). Genome-wide Pleiotropy between Parkinson disease and autoimmune diseases. JAMA Neurol..

[bb0465] Wray N., Visscher P. (2008). Estimating trait heritability. Nature Education..

[bb0470] Wray N.R. (2019). Complex trait prediction from genome data: contrasting EBV in livestock to PRS in humans: genomic prediction. Genetics..

[bb0475] Xue-Shan Z. (2016). Imbalanced cholesterol metabolism in Alzheimer’s disease. Clin. Chim. Acta.

[bb0480] Zhu X. (2015). Meta-analysis of correlated traits via summary statistics from GWASs with an application in hypertension. Am. J. Hum. Genet..

